# Gouty tophus erodes nasal bone

**DOI:** 10.1007/s10067-021-05897-z

**Published:** 2021-10-02

**Authors:** Eva Rottmann, David Bulbin, Anthony Zaklama

**Affiliations:** 1 Department of Rheumatology, Geisinger Medical Center, M.C. 21-52, 100 N. Academy Avenue, Danville, PA 17822 USA; 2 Department of Radiology, Geisinger Medical Center, M.C. 20-07, 100 N. Academy Avenue, Danville, PA 17822 USA

## Presentation

**Fig. 1:**
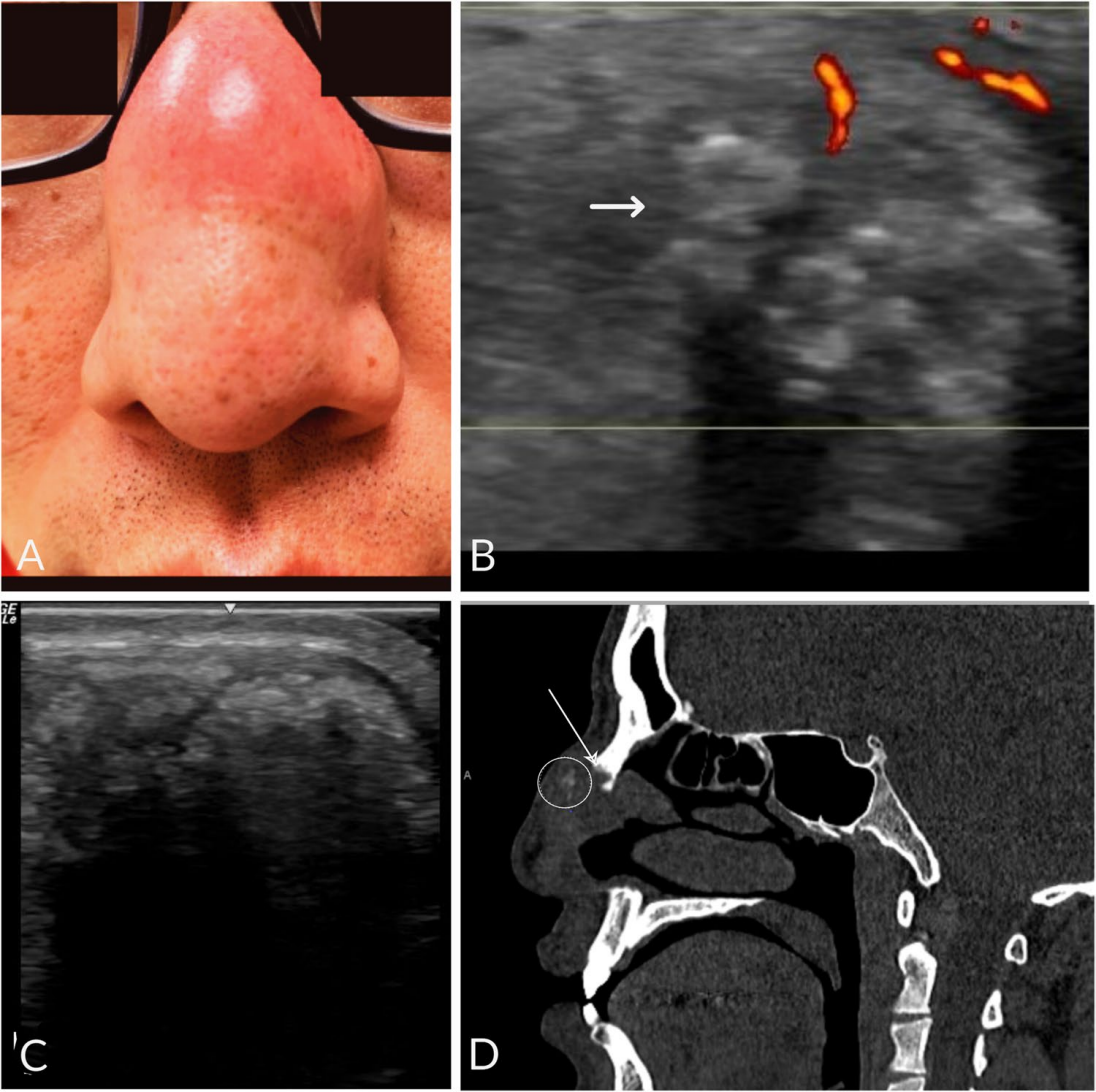
**A** Tender mass over nose. **B** Ultrasound of nasal bridge longitudinal view. **C** Transverse view—an area of inhomogeneous, hyperechoic aggregation with irregular margins, associated shadowing, and a surrounding hypoechoic rim compatible with a “wet clumps of sugar” appearance (white arrow). **D** Sinus CT without contrast shows a soft tissue mass with focal calcifications (circle) immediately adjacent to nasal bone erosion (arrow)

A 45-year-old Asian man with a 20-year history of gout is seen in clinic after lost to follow up for 10 years. He was previously treated with allopurinol but had been off therapy for many years. He describes several weeks of nasal bridge swelling with new episodes of snoring and apnea. On exam, he has a tender, erythematous mass over dorsum of nose with bulky tophi of multiple joints on hands, elbows, knees, and feet. Point-of-care ultrasound of his nasal bridge shows an area of inhomogeneous, hyperechoic aggregation with a hypoechoic rim consistent with gouty tophus [1]. Sinus CT without contrast shows a soft tissue partially calcified mass with bilateral nasal bone erosions. His uric acid was 12.6 mg/dl. This case is a rare presentation of gouty tophi causing nasal bone erosions. The patient tested negative for hereditary enzyme deficiencies. He did test positive for HLA-B*5801 putting him at risk for allopurinol hypersensitivity syndrome. He was treated with febuxostat and eventually pegloticase infusions. With urate lowering therapy, his nasal mass size decreased, and apnea improved without need for surgical intervention (Fig. 1).

## Discussion

Gout is increasingly recognized as a systemic urate deposition disease and can involve extra-articular deposition into soft tissue, tendons, and even cardiovascular tissue [2]. However, gouty tophus with nasal bone destruction is still extremely rare with only a few cases noted in literature [3]. Pathology can confirm monosodium urate crystals [4]. Ultrasound findings of gout such as a double-contour sign (a hyperechoic line of MSU crystals overlying cartilage) or “wet clumps of sugar” are well described in literature and may spare need for invasive tissue confirmation in the appropriate clinical setting [5]. Apneic symptoms can improve with medical treatment alone as nasal tophi decreases; however, some cases of refractory nasal obstruction and septal deviation have required surgical excision [6].
